# The Tale of a Bleeding Tree: A Rare Case of Peripancreatic Variceal Hemorrhage Causing Hemosuccus Pancreaticus

**DOI:** 10.7759/cureus.27106

**Published:** 2022-07-21

**Authors:** Onyinye S Ugonabo, Adnan Elghezewi, Ebubechukwu Ezeh, James Reynolds, Ahmed Sherif, Wesam Frandah

**Affiliations:** 1 Internal Medicine, Marshall University Joan C. Edwards School of Medicine, Huntington, USA; 2 Gastroenterology and Hepatology, Marshall University Joan C. Edwards School of Medicine, Huntington, USA; 3 Interventional Radiology, Marshall University Joan C. Edwards School of Medicine, Huntington, USA; 4 Gastroenterology, Marshall University Joan C. Edwards School of Medicine, Huntington, USA

**Keywords:** complications of pancreatitis, pancreas divisum, intermittent gastrointestinal hemorrhage, gastrointestinal bleed, anemia in chronic pancreatitis, peripancreatic variceal hemorrhage, pancreatitis, peripancreatic varices, hemosuccus pancreaticus

## Abstract

Hemosuccus pancreaticus (HP) is a rare cause of upper gastrointestinal bleeding. It was described by Lawal and Farrel in 1931. This disorder has also been referred to as pseudohemobilia or wirsungorrhagia, caused by bleeding into the pancreatic duct. The rarity of this condition can pose a diagnostic challenge. HP is life-threatening and requires immediate attention. The commonly used treatment modality is coil embolization. Surgery is considered in the case of failed embolization or uncontrolled bleeding. Described below, is a case of a 72-year-old female with a history of chronic pancreatitis who presented with anemia secondary to bleeding peripancreatic varices.

## Introduction

HP has been shown to cause obscure gastrointestinal bleeding in about one in 1500 cases [1]. It can occur in both males and females. The male-to-female ratio is estimated at 7:1, occurring mostly in the fifth to sixth decades of life [2]. There is a wide distribution with the age of onset. The mean age of onset is however between 50 to 60 years [2,3]. HP is predominantly seen in the settings of chronic pancreatitis, pancreatic pseudocyst, pancreatic tumor, pancreatic divisum, and iatrogenic injury to the pancreas. The most common source of bleeding is ruptured arterial pseudoaneurysm which occurs in 3.5% to 10% of pancreatitis [4]. The splenic artery has been reported to be the most commonly involved, followed by gastroduodenal, pancreaticoduodenal, and hepatic artery. Diagnosing HP as a cause of gastrointestinal bleeding can be very challenging because of the anatomy of the bleeding site which is not usually seen during endoscopy. The mortality rate in untreated cases can be up to 90% and ranges between 25% to 37% in treated cases [5,6].

## Case presentation

**Figure 1:**
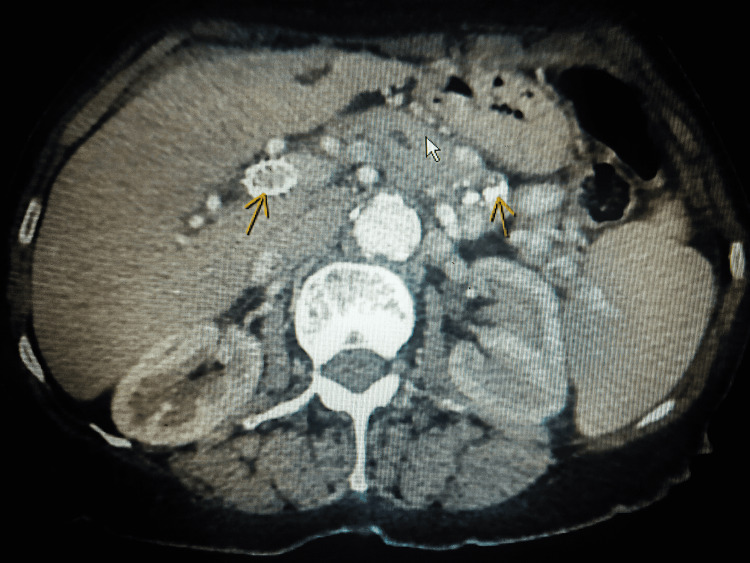
Contrast-enhanced computed tomography (CECT) of the abdomen showing patent TIPS (arrow on the left), dilated pancreatic duct (white arrow), areas of calcification in the pancreatic head (arrow on the right)

**Figure 2:**
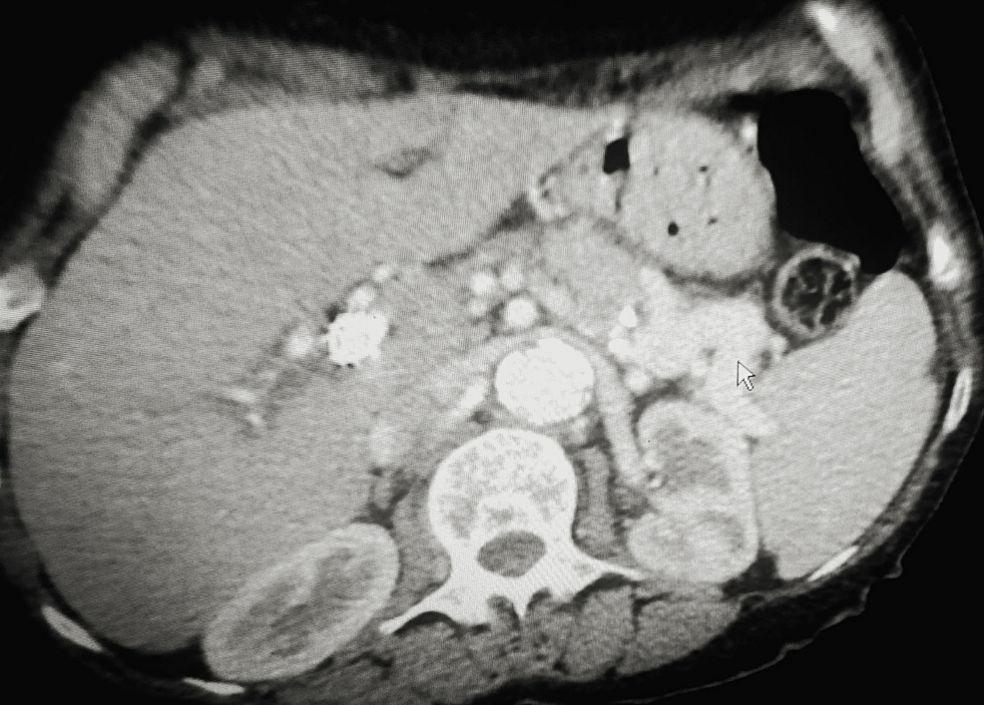
Contrast-enhanced computed tomography (CECT) of the abdomen showing dilated splenic vein (white arrow)

**Figure 3:**
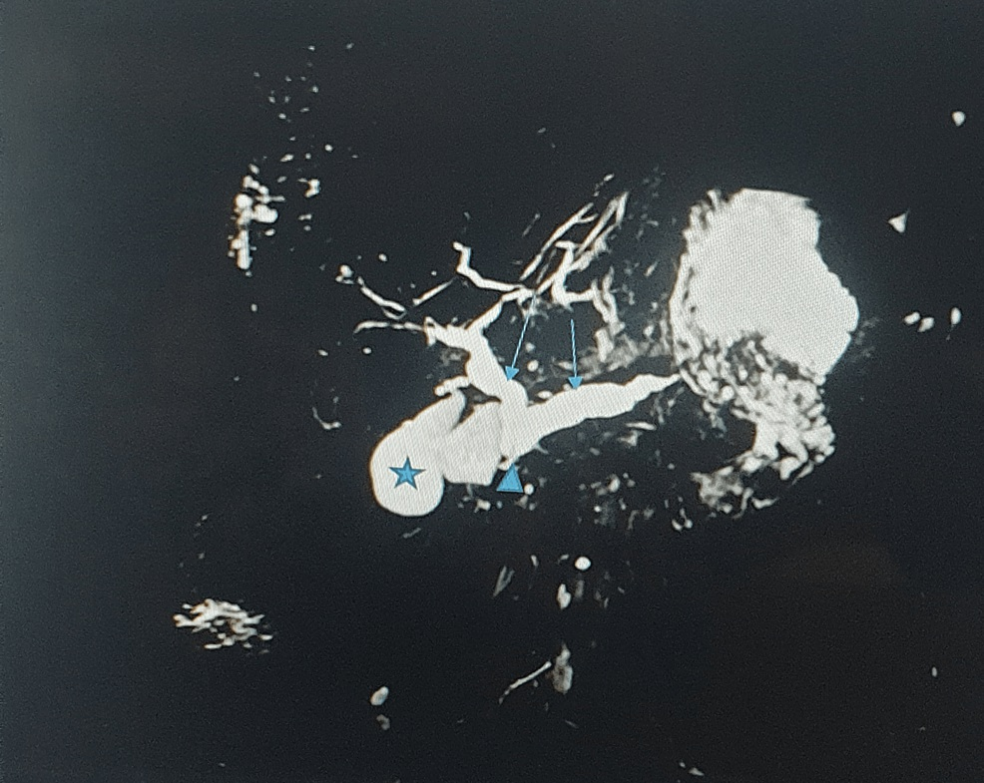
Magnetic resonance cholangiopancreatography (MRCP) showing incomplete pancreas divisum: dilated common bile duct (left arrow), dilated dorsal pancreatic duct (right arrow) draining into the minor papilla (triangle), gall bladder (star).

**Figure 4:**
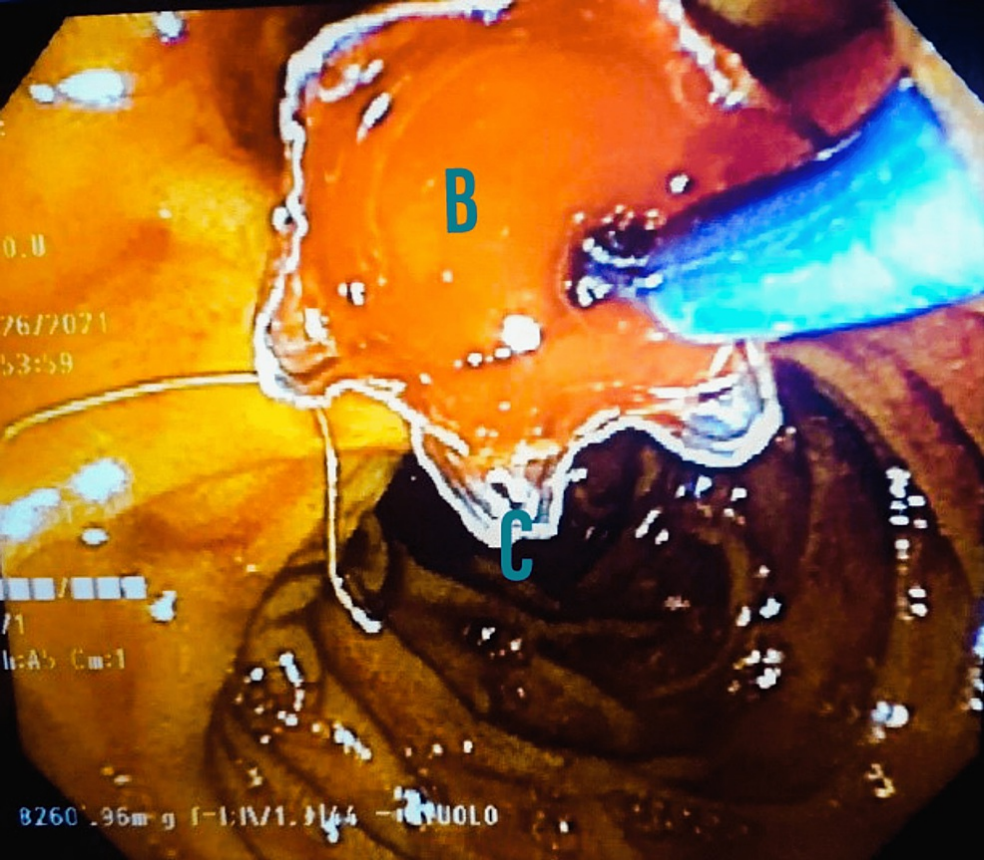
Esophagogastroduodenoscopy (EGD) with endoscopic retrograde cholangiopancreatography (ERCP) procedure showing a metallic stent (C) placed through the bleeding minor papilla (B) into the duct

**Figure 5:**
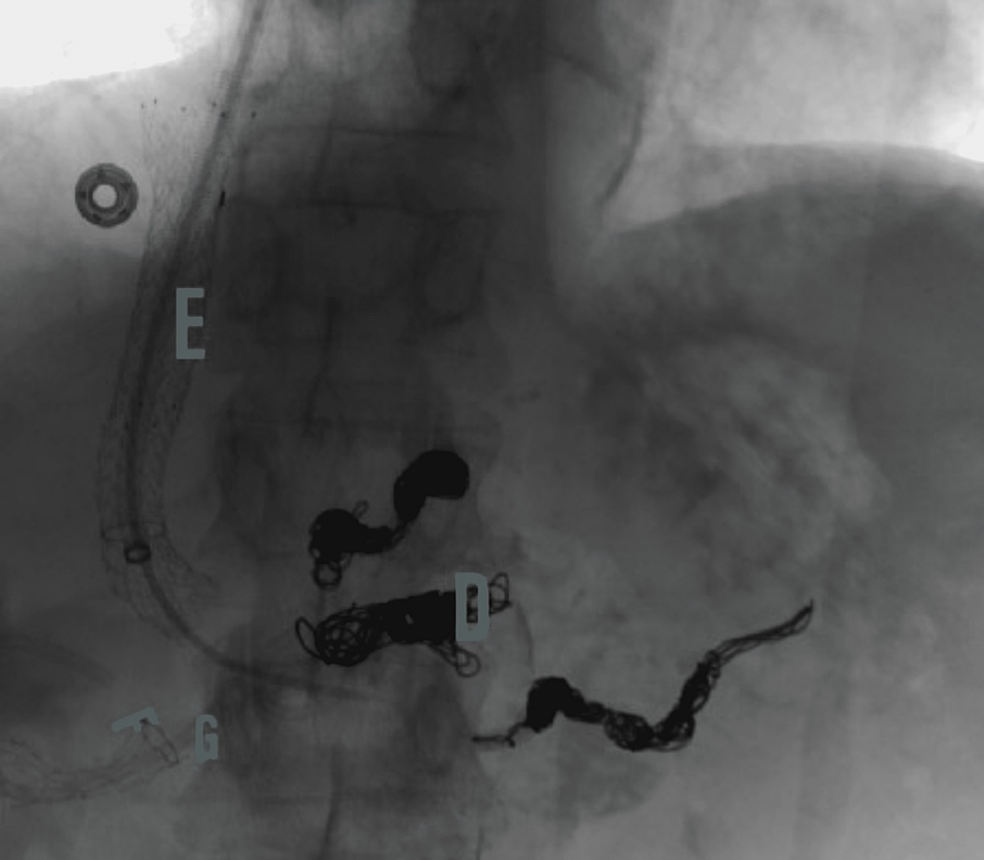
Shows embolized peripancreatic varices (D), a metallic stent (F) transversed with a double pigtail catheter (G) into the main pancreatic duct from the minor papilla. E represents Transjugular intrahepatic portosystemic shunt (TIPS)

A 72-year-old female presented to the emergency department with intermittent epigastric pain that radiates to the back for a week duration. The pain was sharp and worsened with food intake. She denied any history of hematemesis or melena. Her past medical history was significant for chronic pancreatitis with recurrent hospital admissions, and decompensated liver cirrhosis status post-transjugular intrahepatic portosystemic shunt (TIPS). Examination findings were benign except for a body mass index of 17.8kg/m2 (18.5-24.9) and epigastric tenderness. Significant lab findings included white cell count of 11.5k/cmm (reference range 4.5-10), hemoglobin of 13.6k/cmm (12-16), aspartate aminotransferase (AST) of 25 Unit/L (15-37), alanine aminotransferase (ALT) of 20 Unit/L (7-56), alkaline phosphatase (ALP) of 73 Units/L (15-37), Cr of 0.67mg/dl (0.7-1.3), lipase of >11,000Units/L (73-393), total bilirubin of 1.8mg/dl (0.2-1.0), triglyceride of 96mg/dl (10-150). The patient was started on lactated ringers at 250cc/hr. Abdominal ultrasound obtained showed 8mm ductal dilatation, and no gallstone was seen. Contrast-enhanced computed tomography of the abdomen (CECT) showed patent TIPS with a dilated proximal pancreatic duct, calcified pancreatic head, and dilated splenic vein, (Figures 1, 2). Gastroenterology was consulted for endoscopic retrograde cholangiopancreatography (ERCP) due to > 6mm bile duct dilatation with hyperbilirubinemia. The patient however deferred the procedure as abdominal pain significantly improved after 24 hours and she requested to be discharged home and scheduled for an outpatient ERCP. However, she presented to the hospital after four days with similar epigastric pain, dark stool, and inability to keep anything down due to nausea. Vital signs were stable with benign examination findings. Significant laboratory findings include hemoglobin of 9k/cmm, platelet of 108k/cmm, AST of 18Units/L, ALT of 12 Units/L, total bilirubin of 1.6mg/dl, ALP of 46 Units/L and creatinine of 0.4mg/dl. Considering pancreatic duct dilatation seen in the previous admission, magnetic resonance cholangiopancreatography (MRCP) was pursued which showed a pancreas divisum (Figure 3). The patient’s current drop in hemoglobin with melena raised suspicion of a gastrointestinal hemorrhage. Esophagogastroduodenoscopy with endoscopic ultrasound (EGD-EUS) showed blood in the stomach and duodenum due to bleeding from the minor papilla. ERCP revealed dilated pancreatic duct up to 11mm with blood clots inside the pancreatic duct, blood in the minor papilla with peripancreatic varices, and the major papilla appeared normal. Minor papilla cannulation was done and contrast injected showed marked dilatation of the main pancreatic duct up to 14mm with a large filling defect consistent with blood clots. A minor papillary sphincterotomy was done to enlarge the ductal opening. A 10mm x 4cm metallic-covered stent was then placed across the minor papilla into the duct, transversed by a pigtail stent, (Figure 4). A pancreatic ductal sweep was done with the removal of blood clots. The patient was started on octreotide, a proton pump inhibitor drip, and 100mg of rectal indomethacin was given to prevent post-ERCP pancreatitis. The interventional radiology team used ultrasound guidance and placed a 10 French sheath into the internal jugular vein. Catheter and guidewire were used to assess the right TIPS and advanced into the portal venous aspect of the stent. A portal venogram showed perisplenic vein collaterals with peripancreatic varices. Access was gained into these varices with a catheter and coil embolization was performed, (Figure 5). Post embolization CT confirmed successful coil embolization. Abdominal pain improved and the patient was discharged home and scheduled for stent removal in three months. Five weeks later, she reported no abdominal pain on a follow-up visit, and her hemoglobin remained stable.

 

## Discussion

Pancreatitis is an inflammatory condition that destroys endocrine and exocrine pancreatic tissues. About 80% of cases of HP have been associated with acute or chronic pancreatitis as a risk factor [6]. The annual incidence of chronic pancreatitis is estimated to be about 7-10 per 100,000 people. Due to the anatomical position of the splenic vein as it courses posterior to the pancreas, the inflammatory process of pancreatitis can result in thrombosis of the splenic vein and its tributaries including the portal and superior mesenteric vein. This can result in portal hypertension with the formation of peripancreatic varices. We could not identify any splenic vein thrombosis in our patient but she had dilated splenic vein which might have contributed to the formation of the splenic vein collaterals with peripancreatic varices. One of the common complications of pancreatitis is hemorrhage caused by erosion of a major peripancreatic vessel or the rupture of an arterial pseudoaneurysm. Gastrointestinal (GI) bleeding in the setting of chronic pancreatitis can manifest as bleeding from the pancreatic duct as described in our case or from an eroded vessel surrounding a viscera. GI bleed has also been reported in patients with patent TIPS due to malfunction, concurring that shunt patency may not necessarily correlate with clinical features of its dysfunction [7]. Bleeding from HP is usually intermittent and hardly causes hemodynamic instability. The common symptoms encountered by patients with HP include; epigastric pain caused by intraductal obstruction by blood clots resulting in increased ductal pressure, melena, hematemesis, jaundice, vomiting, and weight loss due to fear of eating. Diagnosis remains challenging and is based on findings seen clinically, endoscopically, and radiographically. EGD is one of the initial workups to rule out other possible bleeding sources. It may reveal bleeding from the minor papilla or ampulla of Vater. However, it only detects bleeding from the minor papilla in 30% of the patients [8]. In cases where detection is challenging, a side-viewing duodenoscope can be used. A retrospective study by Yashavanth et al [9] identified overall endoscopic diagnosis to be about 64.4% in patients with HP. CECT is done to detect pseudocyst, aneurysmal lesions, and other pathologies related to the pancreas. Radionuclide red blood cell scintiscan has been used to identify some obscure GI bleeds. Its use however is limited in this case because of the intermittent nature of the GI bleed. ERCP can show the filling defect in the pancreatic duct, identify the source of bleeding, and serves as a means for stent placement. Angiography remains the gold standard diagnostic approach with a sensitivity of up to 96% [2]. Our patient is unique as the source of bleeding was diagnosed with portal venography using the internal jugular vein as the access. Due to the high mortality rate, treatment is recommended immediately after diagnosis. Endovascular procedures commonly done include coil embolization, stent grafting, and balloon occlusion. Coil embolization has a high success rate of 79-100% with low morbidity and mortality [5]. It can serve as a curative or a temporary measure prior to surgery, especially in advanced cases. Our patient had a shunt revision done in addition to the embolization of the peripancreatic varices. Stent graft placement can be used in patients with portal vein stenosis following a serial balloon dilatation and in patients with HP secondary to a pseudoaneurysm of a large vessel. This blocks blood flow towards the aneurysm body. A surgical approach is reserved for patients with failed embolization, and coexisting conditions like gastric outlet obstruction, pancreatic pseudocyst, tumor, or abscess. For those with failed embolization, ligation of the bleeding vessel can be done. Bergert et al [10] however reported a higher rate of rebleeding from this approach and recommended partial pancreatectomy as the preferred choice of treatment. Patients with pseudoaneurysm and pancreatic pseudocyst, benefit from transcystic arterial ligation with cystic drainage, especially for those with bleeding vessels located in the head and body of the pancreas, while distal pancreatectomy is reserved for those with bleeding vessels in the tail of the pancreas [11 ]. Overall, surgical treatment is associated with a success rate of about 85% with a mortality rate between 10-15% [12].

## Conclusions

HP is a rare entity, but given its severity, clinician awareness is important and therefore should be considered a differential diagnosis in patients with a history of chronic pancreatitis presenting with anemia or suspected GI bleeding. This will necessitate immediate consultation of appropriate specialists. Despite the advancement in diagnostic approach available, delay in diagnosis is still common because of the intermittency in a patient's symptoms. The role of emergency surgery for peripancreatic variceal hemorrhage is still debatable. Considering higher mortality and morbidity rates associated with surgery as the initial therapy, recent studies suggest reserving surgical intervention for patients with failed embolization. 
